# Silver Nanowire-Based Flexible Strain Sensor for Human Motion Detection

**DOI:** 10.3390/s24113329

**Published:** 2024-05-23

**Authors:** Abduweli Mijit, Shuo Li, Qiang Wang, Mingzhou Li, Yanlong Tai

**Affiliations:** 1 School of Material Science and Technology, Jiangxi University of Science and Technology, Ganzhou 341000, China; abdwl.mjt@siat.ac.cn; 2 Key Laboratory of Human-Machine Intelligence-Synergy Systems of Chinese Academy of Sciences (CAS), Shenzhen Institutes of Advanced Technology, CAS, Shenzhen 518055, China; s.li3@siat.ac.cn (S.L.); wang0553wuhu@163.com (Q.W.); 3 School of Metallurgical Engineering, Jiangxi University of Science and Technology, Ganzhou 341000, China

**Keywords:** flexible strain sensor, Ag nanowire, motion detection, human–machine interaction

## Abstract

Accurately capturing human movements is a crucial element of health status monitoring and a necessary precondition for realizing future virtual reality/augmented reality applications. Flexible motion sensors with exceptional sensitivity are capable of detecting physical activities by converting them into resistance fluctuations. Silver nanowires (AgNWs) have become a preferred choice for the development of various types of sensors due to their outstanding electrical conductivity, transparency, and flexibility within polymer composites. Herein, we present the design and fabrication of a flexible strain sensor based on silver nanowires. Suitable substrate materials were selected, and the sensor’s sensitivity and fatigue properties were characterized and tested, with the sensor maintaining reliability after 5000 deformation cycles. Different sensors were prepared by controlling the concentration of silver nanowires to achieve the collection of motion signals from various parts of the human body. Additionally, we explored potential applications of these sensors in fields such as health monitoring and virtual reality. In summary, this work integrated the acquisition of different human motion signals, demonstrating great potential for future multifunctional wearable electronic devices.

## 1. Introduction

With the development of material technology [1,2,3], flexible devices are garnering increasing attention in daily applications, such as bendable displays [4], wearable health monitors [5], foldable smartphones, and stretchable E-skins [6]. The burgeoning field of smart wearable devices has become a critical area of research, driving the need for advancements in sensor technology [7]. Sensors are not only required to be lightweight and durable but must also exhibit high sensitivity, which is a prerequisite for accurate motion detection [8,9].

These sensors, which can be classified into resistive [10], piezoelectric [11], capacitive [12], and triboelectric [13] types based on their sensing mechanisms, play a crucial role in detecting tensile force, pressure, and other parameters [14]. Among these, resistive sensors have garnered widespread attention due to their relatively simple working mechanism, feasible fabrication processes, and ease of signal collection [15]. Strain sensors, a type of resistive sensor [16], primarily detect resistance changes due to mechanical stretching [17]. They excel in wearable health monitoring, soft robotics, and human–machine interfaces, providing more intuitive and immersive user experiences [18,19]. These sensors are also valuable in prosthetics, replicating and monitoring natural limb movements with consistent and reliable performance [20,21].

Evaluating strain sensors involves defining parameters such as sensitivity (gauge factor), response time, sensing range, and repeatability [22,23], with high sensitivity and response speed being crucial for accurate motion detection. Despite the development of many strain sensors with high gauge factors (GFs), their construction often relies on expensive materials like graphene, carbon nanotubes, MXene, reduced graphene oxide, and PEDOT:PSS [24]. Moreover, they involve complex manufacturing processes like electrospinning, multi-layer coating, and extensive drying. The complexity and cost of strain sensors have hindered their practical deployment [25]. Additionally, carbon nanotube sensors may face consistency issues during production, resulting in inconsistent sensitivity and conductivity [26,27]. Long-term environmental changes like humidity and temperature can further affect sensor performance [28,29]. While graphene sensors offer good conductivity and flexibility [30], their complex production process and high costs make large-scale production challenging [31], limiting their application in settings where low costs and high sensitivity are needed [32].

In contrast, silver nanowire-based strain sensors demonstrate excellent conductivity and stretchability while offering simpler production processes and lower costs [33], making them a promising material for strain sensors on their own or in combination with carbon nanotubes, graphene, acrylate, and polyurethane [34].

For instance, Xu developed a groundbreaking silver nanowire–eutectic gel strain sensor by embedding silver nanowires within a eutectic mixture, creating a dual-layer conductive architecture. This innovative sensor not only showcased remarkable stretchability and superior sensitivity, with a gauge factor reaching 2.12 × 10^5^ for small strains ranging from 0 to 50%, but also maintained its functionality at low temperatures [35]. Furthermore, leveraging a simple and cost-effective solution-processing method, Ho fabricated another strain sensor from a composite film comprising “soft” gold nanowires and “hard” silver nanowires. This dual-material approach resulted in a sensor that combined high sensitivity, substantial stretchability up to 70%, and optical transparency between 58.7% and 66.7%, alongside the ability to detect extremely subtle strains as low as 0.05% at low voltages of 0.1 V [36].

The choice of polymer substrates and conductive materials significantly impacts the performance of these sensors. Various studies suggest that the interaction between silver nanowires and the substrate material influences the film’s conductivity, transparency, and mechanical properties [37]. Flexible substrates such as thermoplastic polyurethane (TPU), poly(dimethylsiloxane) (PDMS), elastomers, and Ecoflex are typically chosen to provide the required tensile properties [38]. During the production of strain sensors, the drop-casting method is esteemed due to its simplicity and cost efficiency [39], and PDMS is selected as a flexible substrate due to its notable elastic tensile properties and favorable biocompatibility [40,41].

When tracking various human movements, an extensive sensing range is not always necessary, as the extent of tensile deformation experienced by a sensor, such as in facial expressions, laryngeal movements, finger gestures, and elbow bending, is typically limited [42]. An adequate sensing range should be sufficient to effectively monitor human activities without compromising sensitivity [43]. Therefore, the key attributes to consider are the sensor’s sensitivity and response speed [44].

In this study, we aimed to leverage the significant performance and application advantages of silver nanowire-based strain sensors. By optimizing their exceptional electrical conductivity, flexibility, and sensitivity through a practical manufacturing process like drop-casting, we intended to create robust, lightweight sensors capable of capturing subtle resistance changes resulting from physical movements. Adjusting the concentration of AgNWs enabled us to tailor the gauge factor, allowing the sensors to adapt to various types of movement while retaining their functionality, even after 5000 stretching cycles. These sensors offer a reliable solution for a wide range of applications, ranging from wearable health monitoring and virtual reality applications to soft robotics and prosthetics. Ultimately, this research is intended to bridge the gap between high-performance motion detection and practical implementation, providing a solid foundation for developing next-generation motion sensors in wearable electronic devices.

## 2. Experimental Section

### 2.1. Materials and Instruments

A silver nanowire solution (5 mg/mL, 40 nm in diameter, and 30 µm in length) was procured from Hunan NanoUp Printed Electronics Technology Co., Ltd. (Changsha, China). The PDMS base and curing agent were both the Sylgard 184 type and were supplied by Dow Corning (Midland, MI, USA). Anhydrous ethanol (99.7% purity) was obtained from Chongqing Chuandong Chemical (Group) Co., Ltd. (Chongqing, China). All chemicals were used as received without further purification.

The properties were characterized by scanning electron microscopy (SEM, JEOL JSM-7500 F, Tokyo, Japan), and a tensile test was carried out by an MTS C43.104 electronic universal testing machine produced by Metus Industrial Systems (Eden Prairie, MN, USA).

The surface treatment of the substrates was performed using a Nordson MARCH Plasma Series device (Nordson Corporation, Westlake, OH, USA), ensuring enhanced adhesion properties.

Ultrasonic dispersion was facilitated by a Branson SFX250 Sonifier (Emerson Electric Co., St. Louis, MO, USA), providing uniform nanowire dispersion.

The curing and drying processes were executed in a Binder FED Series laboratory oven (Binder GmbH, Tuttlingen, Germany), ensuring precise temperature control.

Additionally, electrical resistivity measurements were conducted using a four-point probe tester (model RTS-8 from Guangzhou Four-Probe Technology Co., Ltd., Guangzhou, China) to evaluate the conductive properties of the AgNW coatings.

### 2.2. Preparation of AgNW Strain Sensors

**Figure 1:**
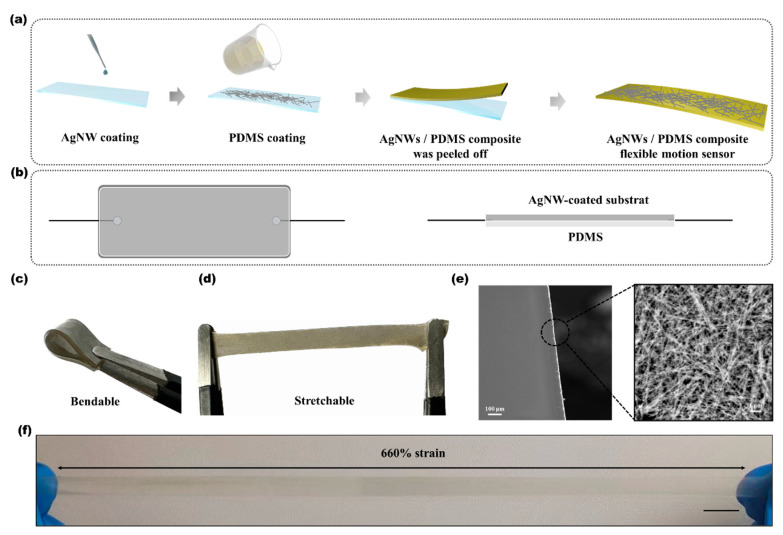
Fabrication and characterization of the AgNW/PDMS composite sensor. (**a**) Fabrication process schematic for the flexible AgNW/PDMS sensor. (**b**) Diagram illustrating the electrode connections within the sensor. (**c**,**d**) Images demonstrating the sensor’s bendability and stretchability. (**e**) SEM images of AgNWs at scales of 100 μm and 1 μm. (**f**) Optical image of the sensor extended to 660% strain, with a scale bar of 10 mm.

The fabrication of the silver nanowire composite flexible strain sensors, as illustrated in Figure 1a, involved several steps. Initially, a PDMS base was prepared by mixing a PDMS oligomer with a curing agent at a ratio of 10:1. This mixture was thoroughly stirred and then degassed in a vacuum chamber to eliminate any air bubbles, ensuring a homogeneous mixture. The degassed PDMS pre-polymer was subsequently either poured into molds or coated onto substrates and cured, resulting in a flexible and transparent base with excellent mechanical properties.

Following the base preparation, silver nanowires were dispersed in an anhydrous ethanol solution using polyvinylpyrrolidone (PVP) and sodium dodecylbenzenesulfonate (SDBS) as dispersants. This step prevented agglomeration and ensured a uniform distribution of nanowires, which were crucial for enhancing the final conductivity and flexibility of the sensor. The solution was stirred and subjected to an ultrasonic treatment to achieve a homogeneous dispersion. The uniformly dispersed AgNWs were then mixed with the PDMS pre-polymer, degassed under vacuum to remove air bubbles, and poured into the molds.

The composite was cured in a temperature-controlled oven at 60 °C for 4–6 h, solidifying into a flexible conductive matrix. After curing, the material was cut to the desired dimensions. Electrical contacts were established by applying high-conductivity silver paste to attach electrodes directly to the ends of the AgNW-coated substrates, as depicted in Figure 1b. Once the paste had cured, fine copper wires were carefully soldered to the electrode terminals using a low-temperature soldering process. This minimized potential damage to the substrate and the AgNW layer.

To ensure durability and maintain functionality under operational stress, such as stretching up to 660% strain, the entire electrode area was encapsulated with a thin layer of PDMS. This encapsulation provided mechanical protection and prevented degradation from environmental factors like moisture and bending, thereby safeguarding the sensor’s integrity and performance in various applications.

## 3. Results and Discussion

### 3.1. Structure and Sensing Properties of Strain Sensor

The fabrication process depicted in Figure 1a effectively integrated silver nanowires into a flexible sensor architecture, confirming the practicality of mass-producing highly aligned and integrated nanowire networks. The resulting sensor demonstrated robust stretchability and flexibility, as illustrated in Figure 1c,d, which are essential attributes that support its broad applicability. It could endure substantial deformation while maintaining full functionality, and its versatility enabled effortless conformance to various shapes and textures.

At higher magnifications, the scanning electron microscope (SEM) images (Figure 1e) revealed detailed insights into the sensor’s nanostructure, displaying a uniform dispersion of nanowires throughout the polymer matrix—a reflection of the precise fabrication techniques employed. Optical images following a 660% elongation (Figure 1f) illustrated the sensor’s exceptional durability, maintaining its structural integrity and conductivity even under extreme stretching.

**Figure 2:**
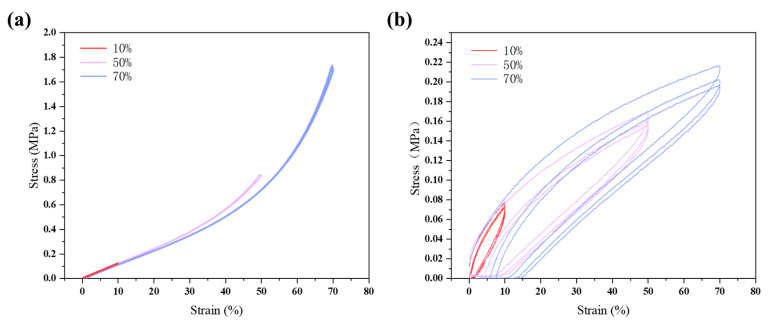
Comparative analysis of cyclic stretching responses of strain sensors on PDMS and PEA substrates. (**a**) Strain responses of sensors on a PDMS substrate at incremental strain levels (10%, 50%, and 70%). (**b**) Strain responses of sensors on a PEA substrate at identical strain levels (10%, 50%, and 70%).

To assess the impacts of different substrates on AgNW sensors, we conducted a comparative analysis of PDMS and PEA substrates, as illustrated in Figure 2. The PDMS substrate demonstrated a prominently elastic behavior at lower strains (10%), with an almost fully elastic response allowing it to revert to its original shape upon load removal. Conversely, the PEA substrate exhibited commendable elasticity at the same strain level but displayed increased hysteresis at higher strains, suggesting the onset of plastic deformation.

Subtle indications of plastic deformation were observed in the PDMS curve at strains of 50% and 70%, where a nonlinear growth pattern suggested material hardening under higher strain. In contrast, the PEA substrate revealed greater hysteresis at these strain levels, implying that it could not fully return to its original shape after cyclic loading, which was indicative of more pronounced plastic deformation.

In terms of fatigue characteristics, the PDMS substrate remained consistent throughout repeated loading and unloading cycles, reflecting superior fatigue resistance. Conversely, the curve for the PEA substrate changed noticeably with each cycle, hinting at potential structural changes within the material after cyclic strain, leading to reduced fatigue performance.

The durability and reliability of the PDMS substrate were further demonstrated by its higher endurance in testing, making it more suitable for long-term applications under sustained loading conditions. While the PEA substrate may perform adequately in short-term high-strain conditions, its performance is likely to degrade under long-term cyclic loading.

Synthesizing these observations, sensors based on the PDMS substrate outperformed those based on PEA in terms of elasticity and fatigue resistance. Although the PEA substrate sensors were adequate for applications requiring high-strain conditions, they exhibited more significant plastic deformation and fatigue over time.

Having established the varied mechanical properties and performances of the PDMS and PEA substrates under different loading conditions, we shifted our focus to the fundamental metrics that gauge the sensitivity of strain sensors. Generally, the sensitivity performances of strain sensors are measured using the gauge factor (GF), which is defined as $GF=(\Delta R/R_{0})/\varepsilon$, where ΔR represents the change in resistance under stretching, $R_{0}$ is the initial resistance, and ε is the applied strain. This measure is crucial for understanding how effectively a sensor can detect and measure strain, particularly under the varied conditions previously described.

To provide a thorough comparison of the gauge factors (GFs) observed in our sensors and those reported in the existing literature, we compiled a detailed table of GF values across various studies. This table is available in the Supplementary Information (Table S1). It offers insights into the sensitivity performance of our sensors relative to similar technologies, facilitating a deeper understanding of their potential applications and limitations. For a complete review of these comparative data, readers are encouraged to refer to Table S1 in the Supplementary Information. The enhanced understanding gained from this comparison enriches the subsequent discussion of the sensor’s operational dynamics, allowing us to draw more nuanced conclusions from the empirical data presented.

**Figure 3:**
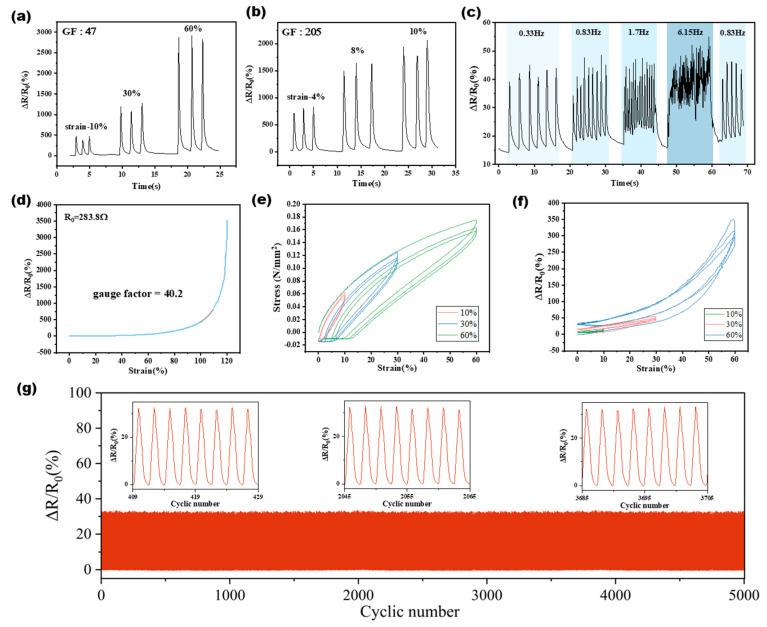
Characterization of the AgNW strain sensor. (**a**) Resistance variation rates examined at strain levels of 10%, 30%, and 60% with a gauge factor (GF) of 47. (**b**) Resistance changes measured at strain levels of 4%, 8%, and 10% for a GF of 205. (**c**) Resistance variation rates analyzed across different motion frequencies (0.33 Hz, 0.83 Hz, 1.7 Hz, 6.15 Hz, and 0.83 Hz). (**d**) Initial resistance quantified at 283.8 Ω, with the application of a tensile testing machine resulting in a GF of 40.2. (**e**) Stress–strain curves plotted for various applied strain levels (10%, 30%, and 60%). (**f**) Resistance variation rates observed for different levels of applied strain (10%, 30%, and 60%). (**g**) Resistance changes recorded after 5000 cycles of reciprocal stretching.

With the gauge factor serving as a foundational metric for assessing sensor sensitivity, we proceeded to scrutinize the specific behaviors and performance characteristics of the AgNW sensor under various operational scenarios, as systematically recorded in Figure 3.

The sensor displayed remarkable sensitivity across varying strain levels, starting with an initial gauge factor (GF) of 47, which clearly highlighted its precise responses to strains of 10%, 30%, and 60% (as shown in Figure 3a). At higher strain levels, the sensor’s sensitivity further intensified, reaching a GF of 205, and the sensor demonstrated minimal resistance changes at strain levels of 4%, 8%, and 10% (referenced in Figure 3b), affirming its utility in applications that demand precise strain detection.

Dynamic performance assessments revealed consistent resistance variations under diverse motion frequencies (illustrated in Figure 3c), effectively demonstrating the sensor’s capability to operate reliably across a spectrum of dynamic conditions, from 0.33 Hz up to 6.15 Hz.

Mechanical durability was rigorously evaluated through tensile testing, which established a GF of 40.2 when the initial resistance was recorded at 283.8 Ω (detailed in Figure 3d). The accompanying stress–strain curves, plotted for applied strains of 10%, 30%, and 60%, showcased the sensor’s robust mechanical properties and its ability to adapt to significant deformations without a loss of functionality (shown in Figure 3e).

Moreover, the sensor exhibited exceptional long-term stability, as evidenced by the minimal resistance changes observed after 5000 cycles of reciprocal stretching (depicted in Figure 3g). This demonstrated its potential for durable and reliable operation in practical applications, confirming its suitability for long-term usage under repetitive strain conditions.

To develop sensors capable of precisely detecting a diverse range of human motions, this study thoroughly investigated the complex relationship between the concentration of silver nanowires (AgNWs) and their electrical impedance.

**Figure 4:**
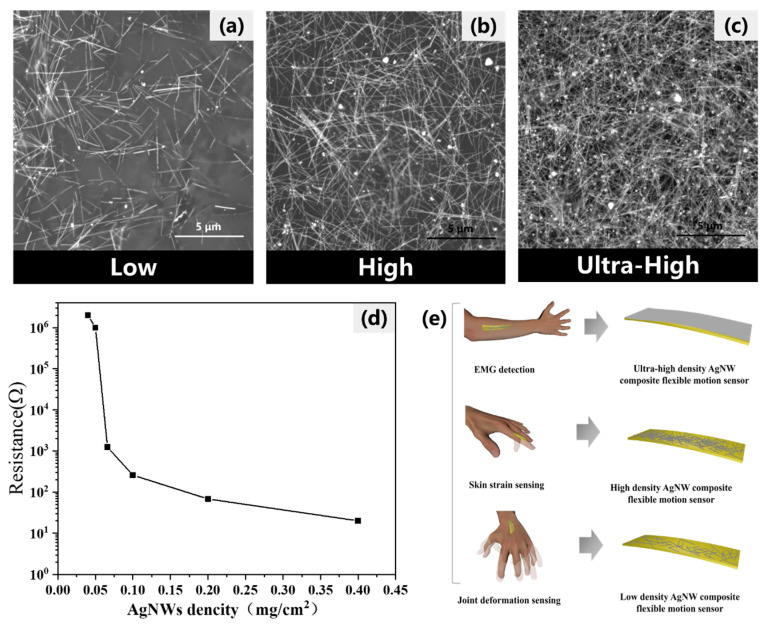
Correlation of AgNW surface density with sensor performance in human motion signal detection. (**a**–**c**) SEM images displaying AgNWs at varying densities from low to ultra-high, captured at a scale of 5 μm. (**d**) Graph illustrating the relationship between the surface density of silver nanowires and the corresponding sensor resistance. (**e**) Illustration of how different AgNW densities are specifically utilized to detect distinct human motion signals.

The optimization of the AgNW density is critical for fabricating sensors that can accurately detect a variety of human motion signals. The SEM images in Figure 4a–c illustrate the progression of AgNW networks from a sparse distribution at a low density, as shown in (a), to a compact arrangement at an ultra-high density, as depicted in (c). These images clearly demonstrate the direct link between increased AgNW density and the formation of a more contiguous conductive network, which is associated with decreased resistance. Figure 4d quantifies this relationship, identifying the saturation point beyond which additional density does not further reduce resistance.

Moreover, Figure 4e explores the practical implications of these varying AgNW densities, demonstrating how different densities are specifically engineered to detect distinct types of human motion. Sensors with lower densities are shown to be effective for broad, general movements, while higher densities excel in capturing subtle and refined motions, such as precise finger movements and facial expressions. This capability is crucial for advanced applications in wearable technology and medical diagnostics, where accurate motion detection is essential.

**Figure 5:**
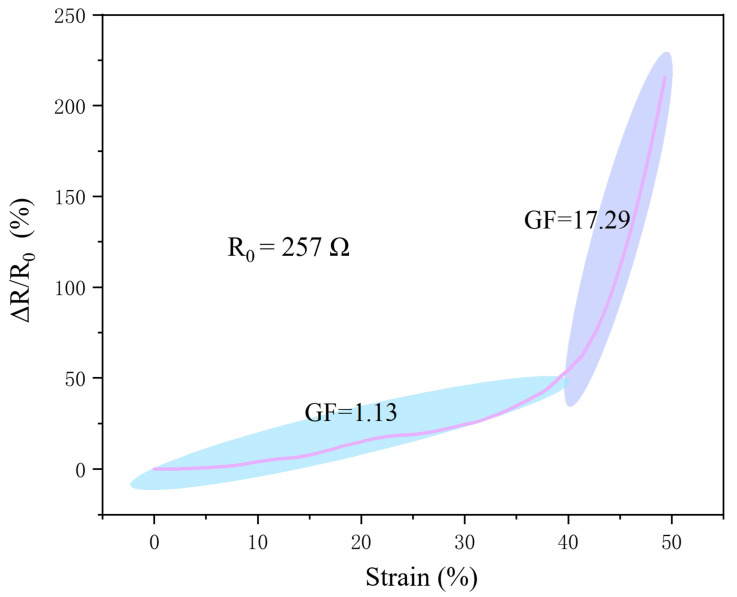
Gauge factors and resistance responses of AgNW-based flexible strain sensor under varying strains.

Figure 5 encapsulates the quintessential findings of the resistance responses of silver nanowire-based flexible strain sensors under mechanical deformations. This graph illustrates how resistance varies with strain (%), a critical factor in the sensory performance and application spectrum of these devices. The curve begins with a near-linear ascent, demonstrating a proportional increase in resistance change as the strain increases from 0% to approximately 20%. During this initial phase, the sensor exhibits a relatively modest gauge factor of 1.13, indicative of a less sensitive response to mechanical deformation. This characteristic is suitable for applications where stability is prioritized over high sensitivity, such as in monitoring postural alignment or detecting gradual movements.

As the strain exceeds the 20% threshold, a distinct inflection point on the curve marks a significant transition in the sensor’s sensitivity. The resistance response curve steepens dramatically, reaching a GF of 17.29, highlighting the sensor’s enhanced ability to detect rapid and extensive deformations. This heightened sensitivity in the high-strain domain is crucial for applications requiring precise monitoring of dynamic movements, such as in high-precision athletic performance tracking or in biomedical settings where large physiological motions need accurate detection.

### 3.2. Sensing Mechanism Analysis

**Figure 6:**
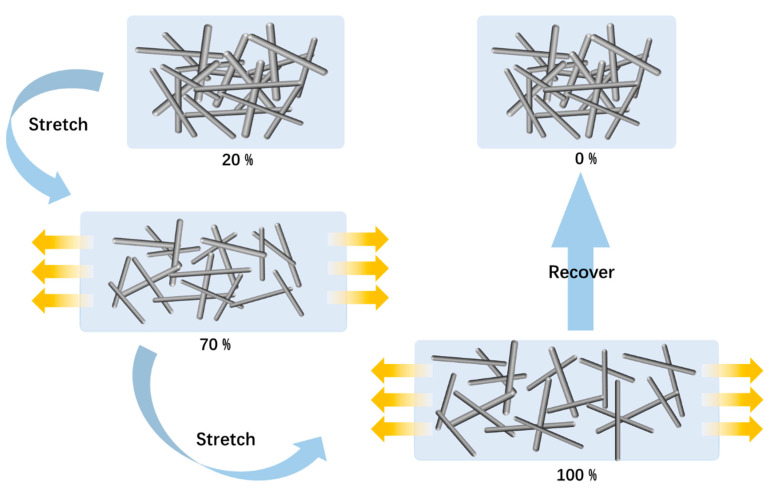
Simulation diagram of the sensing mechanism of the AgNW flexible strain sensor.

An analytical exploration of the silver nanowire-based strain sensor’s conductivity revealed its adaptive responses to varying degrees of strain. Figure 6 shows the strain-sensing mechanism of the AgNW/PDMS flexible strain sensor. This figure demonstrates the working mechanism of the silver nanowire flexible strain sensor under strain.

Initially, at 0–35% strain, the silver nanowire network, acting as the sensor’s conductive core, begins to attenuate, diminishing in width and generating minute fissures perpendicular to the applied stress. This results in a gradual escalation in electrical resistance.

Progressing to the 35–75% strain range, increments in both the number and width of the aforementioned fissures are observed, causing an expedited surge in resistance. Here, the mechanism of crack propagation comes to the forefront as the principal contributor to resistance amplification.

Beyond 75% strain, reaching up to an extreme 600%, the structural “bridges” within the conductive network fracture entirely, leaving behind isolated “blocks.” This structural disruption marks the final stage of the sensor’s mechanical transformation, underlining its capacity to register and respond to high-strain conditions.

### 3.3. Application of Human Motions and Healthcare Monitoring

While existing motion sensors often struggle with issues such as low sensitivity, limited flexibility, or poor adaptability to different anatomical locations, this study leveraged advanced silver nanowire-based strain sensors designed to overcome these limitations. Based on a refined understanding of sensor structure and sensing mechanisms, our research demonstrated enhanced performance and versatility in real-time monitoring of complex human movements across various physiological sites.

**Figure 7:**
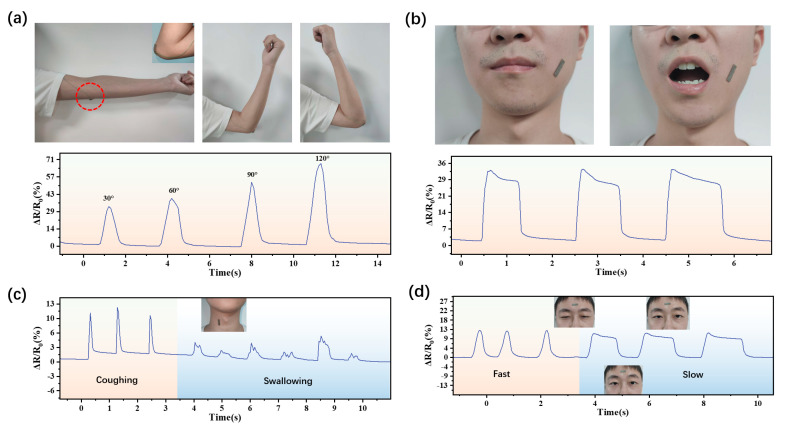
Time–dependent resistance changes in motion sensors attached to various human body parts. (**a**) Sensor attached to the elbow. (**b**) Sensor attached to skin near mouth. (**c**) Sensor attached to the neck. (**d**) Sensor attached to the forehead.

In Figure 7a, the sensor is attached to the elbow to monitor the waveforms of arm-bending movements. The recorded resistance changes accurately depict the flexion and extension of the forearm. Figure 7b showcases the sensor attached to the skin near the mouth, where it is capable of detecting mouth-opening and -closing actions. The resistance changes recorded by the sensor effectively capture oral movements.

When the sensor is attached to a human neck, as shown in Figure 7c, it can effectively detect coughing and swallowing actions. The recorded resistance changes reliably reflect these physiological responses. Figure 7d demonstrates the sensor attached to a human forehead, where it is capable of detecting frowning and eyebrow movements. The measured resistance fluctuations accurately reflect facial muscle contractions.

**Figure 8:**
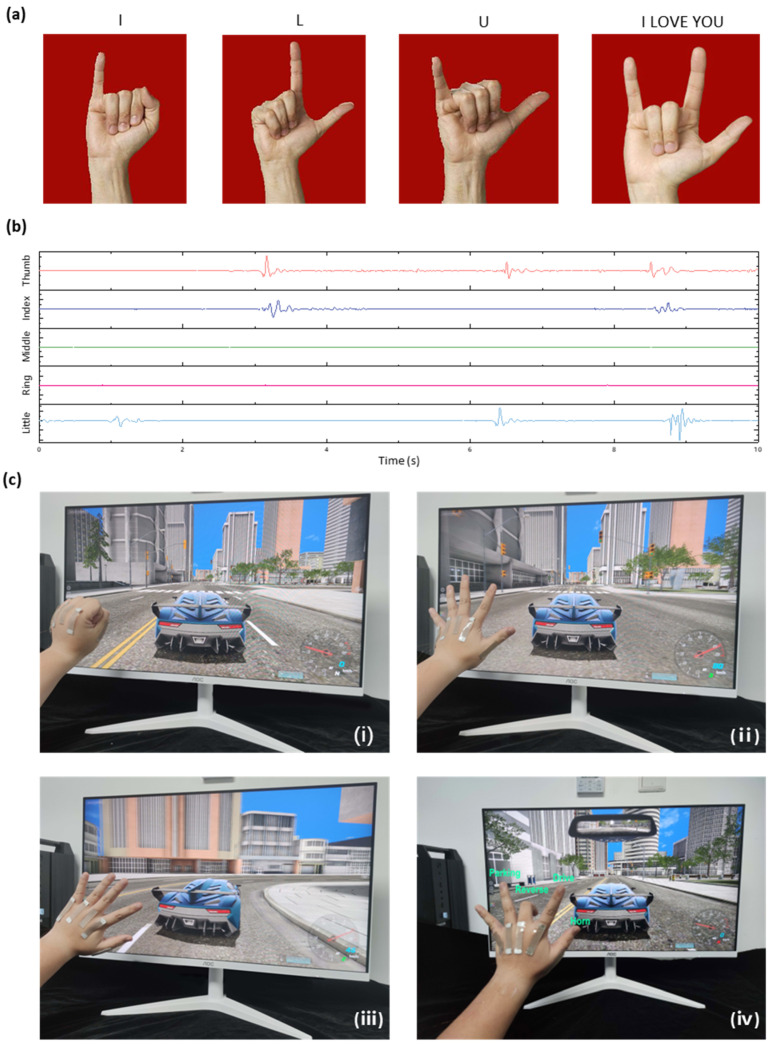
Gesture recognition and virtual reality applications. (**a**) Illustration of “I–L–U (I LOVE YOU)” gestures in sign language. (**b**) Waveform data corresponding to each finger shown in (**a**), detailing the resistance variations during gesture execution. (**c**) Gesture control in a simulated driving scenario: clenching fist to brake (Figure **i**), opening hand to move forward (Figure **ii**), tilting hand for left/right direction control (Figure **iii**), and various hand positions for controlling the car’s modes (parking, reverse, and drive) and honking (Figure **iv**).

These results indicate that when motion sensors are closely attached to different parts of the human body, they successfully capture and interpret specific movements. The sensors exhibit excellent sensitivity and responsiveness to particular actions, enabling precise monitoring and analysis of various physiological and biomechanical activities. Building on this foundational success, our study, as detailed in Figure 8, further extended the application of these versatile sensors to complex communication and interactive environments, demonstrating their efficacy in both sign language recognition and virtual reality (VR) settings.

Figure 8a illustrates the “I–L–U (I LOVE YOU)” gestures in sign language. To capture and analyze these gestures, we recorded waveform data corresponding to each finger’s movement, as shown in Figure 8b. The sensors accurately captured the temporal resistance variations associated with the movements of individual fingers during the execution of sign language gestures.

The waveform data obtained from the sensors provided valuable insights into the dynamics and patterns of finger movements during sign language expression. By analyzing the resistance fluctuations, we were able to distinguish and classify different sign language gestures with a high degree of accuracy.

Expanding the scope, as shown in Figure 8c, we applied the motion sensors in a simulated driving scenario, successfully associating specific hand gestures with various control actions such as braking, moving forward, steering, and controlling the car’s different modes and honking. This application highlights the potential for our sensors to contribute to the development of intuitive and user-friendly control systems.

In conclusion, our results demonstrate the significant potential for motion sensors to advance both sign language recognition systems and interactive control systems. Accurately capturing and interpreting complex movements as waveform data and control actions, respectively, suggests a promising future for the development of more inclusive, interactive technologies.

## 4. Conclusions

In summary, this study successfully engineered a sensor utilizing silver nanowires that demonstrated pronounced stretchability, flexibility, and elasticity and was optimized for human motion detection. This sensor proved its robustness with stable performance over 5000 stretching cycles. It excelled in the nuanced recognition of gestures, converting slight variations in resistance into precise data, which signified its utility in human–machine interaction systems and sign language interpretation. Additionally, its potential for gesture–based control in simulated driving contexts underscores its applicability in advanced human–machine interfaces. This research underscores the pivotal role that AgNW-based sensors could play in enhancing human–machine interaction technologies, and future initiatives will be aimed at refining their manufacturing processes and widening their use in real-world applications.

## Data Availability

Data is contained within the article and Supplementary Materials.

## Supplementary Materials

The following supporting information can be downloaded at: https://www.mdpi.com/article/10.3390/s24113329/s1. Refs. [45,46,47,48,49] are cited in Supplementary Materials.
