# Supplementary material for: Silver Nanowire-Based Flexible Strain Sensor for Human Motion Detection
Source: Sensors (Basel). 2024 May 23;24(11):3329. doi: 10.3390/s24113329 (PMC11174821; doi:10.3390/s24113329)
Supplement: Supplementary file 1 [file sensors-24-03329-s001.zip › sensors-2960640-supplementary.pdf]

Supplementary Information

Table S1. Comparative analysis of gauge factors across different strain sensors.

Reference	Material Description	Gauge Factor (GF)	Performance Insights
This study	Silver nanowires/PDMS	Adjustable (~1.13 to 205)	Highly adaptable GF allowing for diverse applications. Particularly suitable for advanced wearable and health monitoring technologies.
Herrmann et al. [45]	Nanoparticle films	~2 to 5	While cost-effective, these films suffer from low sensitivity, making them unsuitable for applications where fine motion detection is critical.
Nur et al. [46]	Wrinkled ultrathin gold films	~3	Provide flexibility but at the expense of lower gauge factors, which limits their utility in high-precision sensing scenarios.
Correia et al. [47]	Conductive ink (silver ink)	Up to 2.48	Economical for large-scale production but offer only moderate sensitivity, restricting their use in high-performance monitoring.
McManus et al. [48]	Inkjet-printed 2D crystal on paper	Up to 125	Appropriate for flexible and cost-effective applications such as smart packaging, though GF may be insufficient for high-sensitivity needs.
Amjadi et al. [49]	Silver nanowire–elastomer nanocomposite	2 to 14	Highly stretchable, making it ideal for wearable devices; however, its moderate sensitivity may not fit all high-stakes monitoring needs.

45. Herrmann, J., Müller, K., Reda, T., Baxter, G., Raguse, B., Groot, G., Chai, R., Roberts, M., & Wieczorek, L. (2007). Nanoparticle films as sensitive strain gauges. *Applied Physics Letters*, 91, 183105.
46. Nur, R., Matsuhisa, N., Jiang, Z., Nayeem, M., Yokota, T., & Someya, T. (2018). A Highly Sensitive Capacitive-type Strain Sensor Using Wrinkled Ultrathin Gold Films.. *Nano letters*, 18 9, 5610-5617.
47. Correia, V., Caparrós, C., Casellas, C., Francesch, L., Rocha, J., & Lanceros-Méndez, S. (2013). Development of inkjet printed strain sensors. *Smart Materials and Structures*, 22.
48. McManus, D., Vranic, S., Withers, F., Sanchez-Romaguera, V., Macucci, M., Yang, H., Sorrentino, R., Parvez, K., Son, S., Iannaccone, G., Kostarelos, K., Fiori, G., & Casiraghi, C. (2017). Water-based and biocompatible 2D crystal inks for all-inkjet-printed heterostructures.. *Nature nanotechnology*, 12 4, 343-350.
49. Morteza Amjadi et al. "Highly stretchable and sensitive strain sensor based on silver nanowire-elastomer nanocomposite.." *ACS nano*, 8 5 (2014): 5154-63.
